# Can You Tell Me where Wally Is?

**DOI:** 10.1068/ig6

**Published:** 2013-10-01

**Authors:** A D F Clarke, M Elsner, H Rohde

**Affiliations:** University of Edinburgh, UK; Ohio State University, USA; University of Edinburgh, UK

## Abstract

Referring expression generation can be thought of as the converse problem to visual search: given a scene and a target, the participant's task is to generate a description which would allow somebody else to quickly and accurately locate the target. While this problem has been studied in psycholinguistics and natural language processing, we believe that vision science also has a role to play. In particular, previous work on this problem is based on simple scenes consisting of a small number of objects and treats vision almost as a pre-process that extracts feature categories for each object in the scene. However, it is unlikely these models will scale: we know from the visual search literature that some descriptions are better than others at enabling listeners to search efficiently within complex stimuli. We hypothesize speakers will be sensitive to visual features allowing them to compose such ‘good’ descriptions. In the present study, we investigate how visual properties (salience, clutter, area and distance) influence REG using images from the “Where's Wally?” books [Handford 1987], which are an order of magnitude more complex than the stimuli traditionally used in REG experiments. We find that referring expressions for large salient targets are shorter than those for smaller and less salient targets. and that targets within highly cluttered scenes are described using more words. The choice of spatial relations also appears to be influenced by visual properties as participants show a preference for referencing large, salient landmarks that are in close proximity to the target.

